# Role of the RAAS in mediating the pathophysiology of COVID-19

**DOI:** 10.1007/s43440-024-00596-3

**Published:** 2024-04-23

**Authors:** Jakub Jasiczek, Adrian Doroszko, Tymoteusz Trocha, Małgorzata Trocha

**Affiliations:** 1 Department of Cardiology, Regional Specialist Hospital in Wrocław, Kamieńskiego 73a, Wrocław, 51-124 Poland; 2 Department of Cardiology, 4th Military Hospital, Faculty of Medicine, Wroclaw University of Science and Technology, Weigla 5, Wrocław, 50–981 Poland; 3 Faculty of Medicine, Wroclaw Medical University, Borowska 213, Wrocław, 50-556 Poland; 4 Clinical Department of Diabetology and Internal Disease, Wroclaw Medical University, Borowska 213, Wrocław, 50–556 Poland

**Keywords:** Coagulation, COVID-19, SARS-CoV-2, RAAS, Angiotensin, ADAM17

## Abstract

The renin-angiotensin-aldosterone system (RAAS) holds a position of paramount importance as enzymatic and endocrine homeostatic regulator concerning the water-electrolyte and acid-base balance. Nevertheless, its intricacy is influenced by the presence of various complementary angiotensins and their specific receptors, thereby modifying the primary RAAS actions. Angiotensin-converting enzyme 2 (ACE2) acts as a surface receptor for SARS-CoV-2, establishing an essential connection between RAAS and COVID-19 infection. Despite the recurring exploration of the RAAS impact on the trajectory of COVID-19 along with the successful resolution of many inquiries, its complete role in the genesis of delayed consequences encompassing long COVID and cardiovascular thrombotic outcomes during the post-COVID phase as well as post-vaccination, remains not fully comprehended. Particularly noteworthy is the involvement of the RAAS in the molecular mechanisms underpinning procoagulant processes throughout COVID-19. These processes significantly contribute to the pathogenesis of organ complications as well as determine clinical outcomes and are discussed in this manuscript.

## Introduction

The renin-angiotensin-aldosterone system (RAAS) stands among the paramount regulatory systems involved in numerous physiological and pathological processes, including cell growth, regulation of water-electrolyte and acid-base balance, inflammatory responses, and the signaling pathway activation [1]. The intricacy of this system is compounded by the multitude of angiotensins generated through the enzymatic conversion of angiotensinogen and its derivatives, along with diverse specific receptors that modulate the primary actions of RAAS [2, 3].

Angiotensin-converting enzyme 2 (ACE2) acts as a surface receptor on the host cells for certain members of *Coronaviridae*, including SARS-CoV-2. Consequently, it serves as a critical link between the RAAS and COVID-19 infection [4]. While the influence of the RAAS on the progression of COVID-19 has been a topic of debate since the pandemic’s onset, and numerous inquiries have been addressed, the full extent of RAAS involvement in the long-term consequences of COVID-19, such as long COVID and the cardiovascular thrombotic effects post-COVID, along with those following vaccination, remains not entirely comprehended. The role of the RAAS in procoagulant processes during COVID-19 is of particular importance. These processes are responsible for most organ complications and determine the clinical outcomes.

This review aims to offer a closer insight into the current knowledge about pathophysiological connections between RAAS imbalance induced by SARS-CoV-2 infection and the progression of this disease as well as its complications. Additionally, this paper explores both novel and existing COVID-19 treatment strategies focusing on RAAS components. We primarily searched for the articles from the timeframe of 2003 to 2023 within medical databases, including PubMed, Scopus and Web of Science using the following keywords: coagulation, COVID-19, SARS-CoV-2, RAAS, angiotensin, ADAM17, pathophysiology and inflammation.

### ACE2 and SARS-CoV-2

The angiotensin-converting enzyme (ACE) in humans is present in two isoforms - somatic and germinal, which differ in the structure of extracellular domains and distribution in organs [5, 6]. The multiorgan effect of pharmacological inhibition of this enzyme remains incompletely elucidated [7]. The ACE homolog (ACE 2) is a transmembrane glycoprotein present in various organs, including the coronary vessels’ endothelium and the renal tubules’ epithelium [8]. Unlike ACE, ACE2 contains a single catalytic domain with a carboxypeptidase activity and a specific affinity to hydrophobic and/or basic amino acids [9]. It hydrolyses angiotensin I (Ang I), angiotensin II (Ang II), and bradykinin metabolites, however, unlike ACE, does not degrade bradykinin itself [10]. In the reaction catalyzed by ACE2, both Ang I and Ang II are converted to angiotensin 1–9 (Ang 1–9) and angiotensin 1–7 (Ang 1–7), respectively, with beneficial effects on the cardiac muscle [11]. Increased expression of ACE2 has been shown in humans and rats during the development of heart failure and following myocardial infarction [12]. It was proved that ACE2, by converting the Ang II to Ang (1–7), counteracts myocardial fibrosis [13, 14].

ACE2 acts as a functional host cell surface receptor, which facilitates the entry of SARS-CoV-2 into cells [4]. Its presence in various elements of the respiratory, digestive, urinary, and cardiovascular systems suggests the development of their dysfunction in the course of SARS-CoV-2 infection [14–16]. The virus is also likely pathogenic in testicular tissue, expressing concerns about fertility in young men [17].

Coronaviruses belong to the group of single-stranded RNA viruses with positive-sense RNA polarity. They are classified into four groups based on their genetic characteristics, with α and β strains being pathogenic to mammals including humans [18]. Apart from RNA, the virus comprises key structural components, including the nucleocapsid protein, envelope protein, membrane protein, and spike (S) glycoprotein [19]. The S glycoprotein exhibits unique protrusions that give the virus its crown-like appearance on the outer surface. This protein is further divided into two subunits, S1 and S2, which play pivotal roles in the interaction between the virus and the host cell membrane during entry. The protease-mediated cleavage of the S glycoprotein into S1 and S2 subunits enhances its affinity for the host cell surface receptor. Within the S1 subunit, the receptor-binding domain (RBD) undergoes complex dynamic conformational changes, exposing or concealing receptor-binding determinants [20]. The S1 subunit is organized into three domains: A, B, and C [21]. SARS-CoV-2 and SARS-CoV, through the B domain, enter host cells by binding to specific cell surface receptors, such as human angiotensin-converting enzymes: ACE2 and CD90L [22, 23]. Mutations in the receptor-binding sites, particularly in the RBD of the S1 subunit of the S protein and in membrane proteins, interfere with the transition from SARS-CoV to SARS-CoV-2 [24]. It has been proposed that the SARS-CoV-2 S protein exhibits 10-20-fold greater binding affinity to human ACE2 compared to SARS-CoV [25].

ACE2 serves as the specific functional receptor for SARS-CoV-2, facilitating virus attachment and entry into host cells via the spike protein on the virus’s surface. Soluble ACE2 (sACE2) released into the extracellular fluid can bind circulating virions, potentially reducing SARS-CoV-2 infectivity [26]. Paradoxically, plasma sACE2 activity has shown a positive correlation with the severity and mortality of COVID-19 [27]. Animal studies have demonstrated that both SARS-CoV-2 infection and the introduction of the S protein into mice result in reduced ACE2 expression, primarily due to the progression of lung damage [28, 29]. The loss of pulmonary ACE2 function leads to increased Ang II production by the ACE enzyme. Overactivation of the RAAS may contribute to the worsening of lung damage by enhancing the inflammatory response, causing a cytokine storm, and activating the NADH/NADPH oxidase system, which exacerbates vasospasm [28, 30, 31]. Active bradykinin metabolites, des-Arg(9)-BK, and Lys-des-Arg(9)-BK, are also substrates for ACE2. Physiologically, ACE2 serves as a protective factor against pulmonary edema by inactivating these kinins. In the course of COVID-19, decreased ACE2 activity may promote edema, inflammation, and oxidative stress [32, 33]. A mice-model study has demonstrated that ACE2 depletion in the lungs is associated with the activation of des-Arg(9)-BK, a mediator of pneumonia, resulting in a more severe course of pneumonia [33].

ADAM17 (A Disintegrin and Metalloproteinase 17), also known as sheddase, is responsible for cleaving various proteins, including ACE2, leading to decreased expression of membrane-bound ACE2 (mACE2) and increased concentration of sACE2. This, in turn, limits the availability of entry points for viruses, potentially reducing viral infectivity. However, two studies have revealed that sACE2 can also facilitate SARS-CoV-2 cell entry [34, 35]. sACE2 forms complexes with SARS-CoV-2 virions and it is suspected that these complexes can spread to distant tissues, potentially leading to multi-organ failure [36]. . As mentioned previously, a high sACE2 concentration in patient’s serum has been associated with a more severe COVID-19 outcome [27]. However, it has been demonstrated that recombinant human soluble ACE2 (rhsACE2) has the potential to reduce the severity of COVID-19. The rhsACE2 is believed to inhibit mACE2 shedding by blocking binding sites in the RBD of the virus, potentially restoring the local Ang II/Ang 1–7 ratio in favor of the latter. The ACE2/Ang 1–7/Mas receptor (MasR) axis has anti-inflammatory effects, which may reduce the release of damaging cytokines [37]. Some experts have explored strategies such as the production of sACE2-IgG conjugates to enhance the activity and half-life of rhsACE2 against SARS-CoV-2 infection [38].

**Fig. 1:**
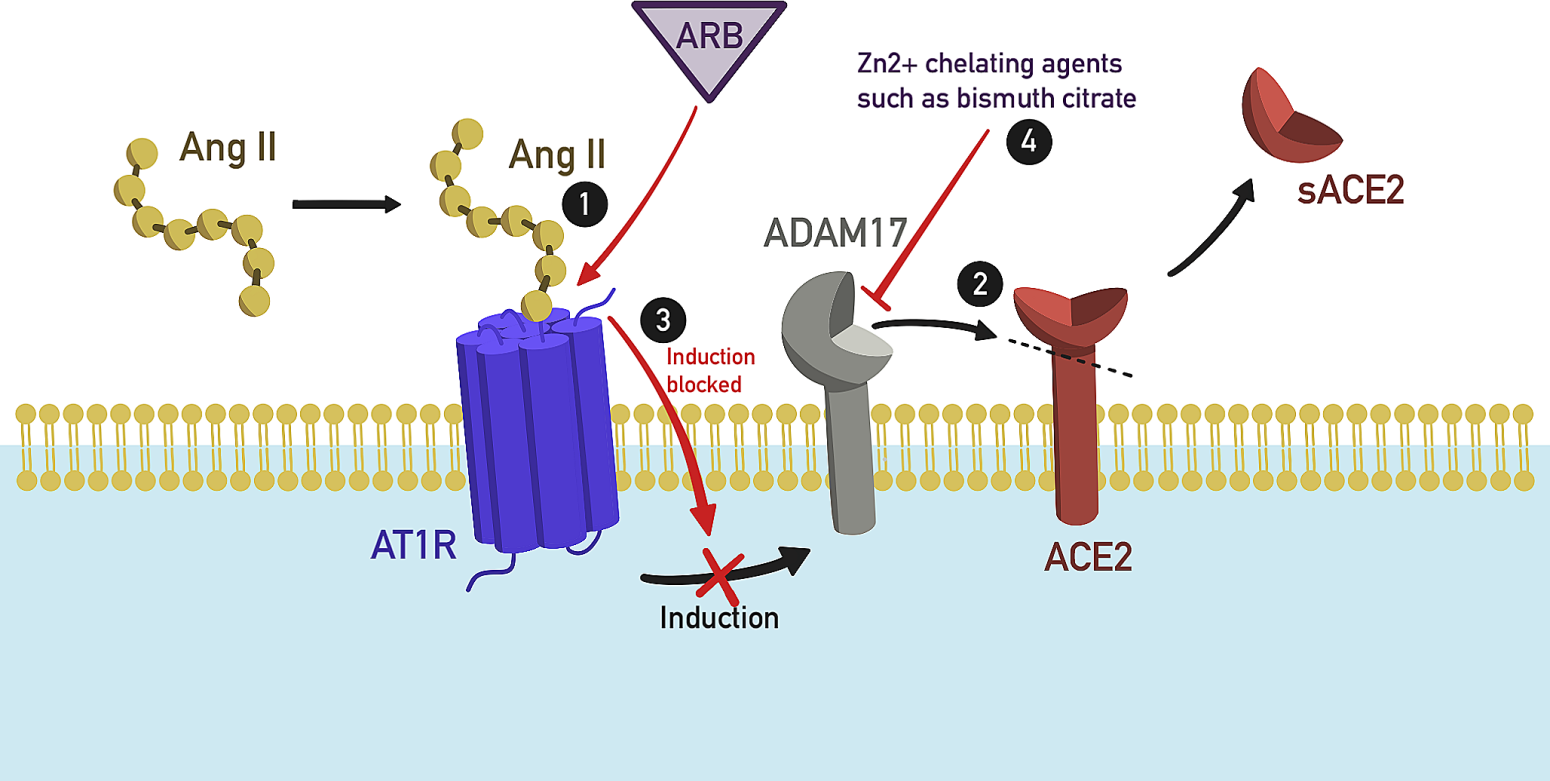
AT1R, ADAM17 and ACE2 interaction. (1) Ang II binds to AT1R, leading to the induction of ADAM17; (2) ADAM17 cleaves ACE2, resulting in the release of sACE2; (3) In presence of ARB, such as losartan, Ang II cannot bind to its receptor and the induction of ADAM17 is stopped. (4) Zn2 + chelating agents block ADAM17 metalloproteinase and inhibit ACE2 shedding. This results in a decrease in circulating sACE2 and increase in mACE2. *Ang II* angiotensin II, *AT1R* angiotensin II receptor type 1, *ACE2* angiotensin converting enzyme 2, *sACE2* soluble angiotensin converting enzyme 2, *ARB* angiotensin II receptor blocker, *ADAM17* A disintegrin and metalloproteinase 17

Ang II has been shown to induce ADAM17 via angiotensin type 1 receptor (AT1R), resulting in the release of the ectodomain of mACE2 into the plasma as sACE2. In this context, the use of AT1 receptor blockers, like losartan, can limit both the decrease in mACE2 and the increase in circulating sACE2 [39–41] (Fig. 1). COVID-19 infection also upregulates ADAM17 expression at both protein and transcript levels [42]. sACE2 levels also increase in pathologies associated with higher AT1R stimulation, such as myocardial infarction, heart failure (HF), metabolic syndrome, and diabetes [37]. Interestingly, when analyzing subjects diagnosed with heart failure with reduced ejection fraction (HFrEF) or with mid-range ejection fraction (HFmrEF), Conti et al. described a significant positive correlation between Sirtuin 1 (Sirt1) activity in peripheral blood mononuclear cells and both circulating ACE2 concentrations and the ejection fraction (EF). High Sirt1 levels may reflect adaptive activation of the sympathetic nervous system and RAAS in systolic HF. Sirt1, which is a NAD+-dependent deacetylase, is highly involved in HF-related processes such as oxidative stress, cell senescence, and energy production as well as in Ang II-induced vascular remodeling [43]. 

However, ADAM17-dependent ectodomain shedding is a saturable process and does not increase when ADAM17 expression exceeds a certain level [44]. While anti-ADAM17 compounds can upregulate mACE2 expression, increasing viral binding to ACE2-positive cells, they can inhibit systemic ACE2 release in the early phase of COVID-19, reducing sACE2 and suppressing viral infectivity [45]. To reduce the activity of ADAM17 and ACE2, compounds capable of chelating Zn2 + ions in the catalytic center of these metalloproteinases have been employed in several studies. Both EDTA and bismuth citrate chelate Ca2 + and Zn2 + ions with varying affinities and exhibit anticoagulant properties [46, 47]. In an animal model, bismuth citrate has shown effectiveness against SARS-CoV-2 [48]. CaNa_2_EDTA, as well as phytates, folic acid, nicotinamide, and zeolites, possess similar properties [46, 47]. Unfortunately, clinical trials evaluating the effectiveness of chelating compounds in combating SARS-CoV-2 infection are currently missing.

ADAM17 undergoes activation through the cleavage of its prodomain by a proprotein convertase, such as furin [49]. This activation process is tightly regulated by various signaling pathways, including phosphorylation of its cytoplasmic domain and interactions with other proteins, such as TIMP3 (tissue inhibitor of metalloproteinase 3). Notably, in vitro studies have demonstrated that serine proteases like furin and TMPRSS2 (cellular transmembrane protease serine 2) can significantly enhance the efficiency of SARS-CoV-2 replication [50]. In a clinical investigation involving a limited cohort of COVID-19 patients, a strong correlation was observed between the circulating furin levels and the severity of the disease [51]. Recent inquiries have uncovered intriguing insights into the virus’s behavior. Beyond its interaction with ACE2, the virus appears to exploit additional receptors or a distinct set of coreceptors, particularly in various organs [52]. For instance, TMPRSS2 plays a pivotal role in activating the S protein and facilitating the fusion between the virus and the plasma membrane, thereby influencing its tropism [53].

Two-thirds of the viral RNA undergo translation, yielding two fundamental polypeptides, while one-third is transcribed into antisense RNA, serving as a template for subsequent replication. Furthermore, antisense RNA exhibits the capability to synthesize several discrete nested (subgenomic) mRNAs of small size, which are then translated to generate structural proteins responsible for nucleocapsid and viral envelope formation. In the final stages of the virus life cycle, viral release from the host cell occurs via exocytosis or fusion with the cell membrane [54].

### Cardiovascular disorders in the course of COVID-19 and of the long COVID

A meta-analysis showed that hypertension, atherosclerotic cardiovascular disease (ASCVD), diabetes (DM), chronic obstructive respiratory disease, cancer and chronic kidney disease (CKD), as well as smoking, were the most common predictors of the need for hospitalization of COVID-19 patients [55] and have been shown to worsen the prognosis. Hypertensive patients more commonly suffered from dyspnoea and comorbidities, had abnormal laboratory tests, and required intensive hospital care [56]. The expected more severe course of COVID-19 in hypertensive patients may have a plausible pathophysiological basis. It is widely recognized that the rapid deterioration in COVID-19 patients is associated with a pro-inflammatory cytokine storm. Consequently, elevated systemic levels of cytokines (e.g., interleukins (IL) - IL-2, IL-6, IL-7, granulocyte colony-stimulating factor (G-CSF), and tumour necrosis factor-alpha (TNF-alpha)) have been noted in individuals with COVID-19. These same cytokines have been scrutinized for their association with the development of hypertension in both, experimental and clinical studies. Lymphocyte count and function serve as common markers. Lymphocyte depletion stands as a prominent feature of COVID-19 infection. Intriguingly, recent evidence suggests a causal relationship between hypertension and lymphocyte count [57].

Cardiovascular events frequently reported in patients suffering from SARS-CoV-2 infection include pulmonary embolism, acute myocardial injury, myocarditis, acute coronary syndrome, pericarditis, and arrhythmias. However, it remains unclear whether these complications result directly from viral damage or arise from hyperinflammation and cytokine storm during the progression of COVID-19. Pulmonary embolism has been associated with viral activity and hypercoagulable state. Contrary, it is proposed that myocarditis, myocardial injury, and certain arrhythmias such as atrial fibrillation may result more from the inflammatory response triggered by the infection rather than from direct involvement of the virus. Interestingly, the COVID-19 pandemic has resulted in a significant increase in the occurrence of stress-induced cardiomyopathy (Takotsubo syndrome), not only among SARS-CoV-2 infected patients but also in the general population [58]. 

Symptoms affecting the cardiovascular system are also common manifestations of long COVID (LC), other known as post-COVID condition or post-acute sequelae of SARS-CoV-2 infection (PASC). According to the World Health Organization (WHO), this condition can be diagnosed in patients who had COVID-19 onset at least 3 months earlier and are affected by new or persistent symptoms for longer than 2 months. Cardiovascular pathologies commonly described as part of long COVID include myocarditis, pericarditis, myocardial ischemia, microvascular dysfunction, non-ischemic cardiomyopathy, thromboembolic disease, arrhythmias, and cerebrovascular events. Other, non-cardiovascular manifestations of long COVID include asthenia, brain fog, cognitive dysfunction, headache, sleep disturbances, taste and smell dysfunctions, gastrointestinal disorders, psychiatric symptoms (anxiety, depression, post-traumatic shock syndrome), alopecia, myalgia, joint pain, skin rashes, low-grade fever, and erectile dysfunction. Dysautonomia is also reported as an important component of long COVID symptomatology, with postural orthostatic tachycardia syndrome (POTS), neurocardiogenic syncope, and orthostatic hypotension being the most prominent manifestations [59]. 

Some evidence suggests that immune system dysregulation, autoimmunity and endothelial dysfunction play pivotal roles in the pathogenesis of these long COVID symptoms. Additionally, viral persistence within various organs remains a critical consideration. It is postulated that immune dysregulation may ensue, possibly due to perturbations in the intestinal microbiota induced by persistent viral presence. Dysbiosis of the intestinal microbiota may also precipitate neurological manifestations. The ability of the spike protein to traverse the blood-brain barrier is noteworthy. Furthermore, the injury inflicted upon endothelial cells by nucleocapsid and envelope proteins triggers cytokine induction and immune activation. Additionally, the prolonged presence of COVID-19 infection and immune dysregulation may lead to the reactivation of other viruses (Epstein-Barr virus, human herpesvirus 6, and cytomegalovirus) and the onset of bacterial diseases (tuberculosis) [60]. It is also suggested that the autonomic nervous system may be directly damaged by the SARS-CoV-2 virus or by an autoimmune process, leading to dysautonomia [58]. 

### Effects of ACEIs and ARBs in COVID-19 patients

A vast majority of patients, including those suffering from COVID-19, are given either angiotensin-converting enzyme inhibitors (ACEIs) or angiotensin AT1 receptor blockers (ARBs) in the management of hypertension, heart failure, or CKD [56, 61]. As is well known, these drugs have a beneficial effect on the cardiovascular system, e.g. by lowering the level of Ang II, but also by inhibiting the decline of ACE2 [62, 63]. Many studies have attempted to clarify whether drugs that increase the activity and expression of ACE2 in animals and humans [64, 65] may adversely affect the prognosis of COVID-19 by facilitating the entry of the virus into host cells [56, 64, 66]. As we already know, there is no evidence to support this hypothesis [67]. In contrast, over-activation of the RAAS was found during COVID-19 infection, resulting in reduced ACE2 activity and increased progression of pneumonia [68, 69]. Therefore, it is suggested that drugs inhibiting the RAAS by increasing ACE2 expression and activity may even protect the lungs from serious damage [30, 70].

**Fig. 2:**
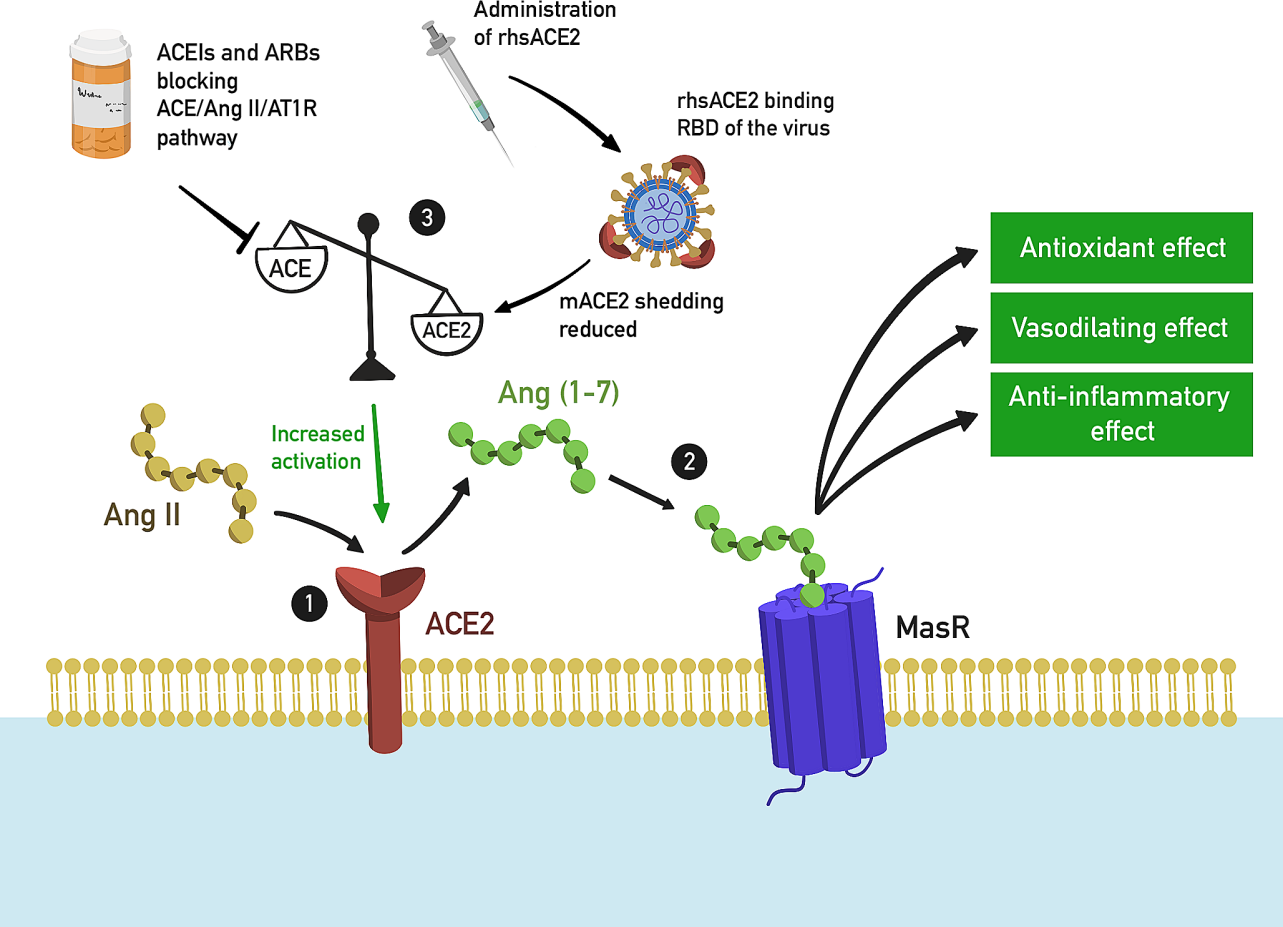
Positive effects of administration of ACEIs, ARBs and rhsACE2. **(**1) ACE2 breaks down Ang II into Ang 1–7; (2) Ang 1–7 binds to the MasR, activating it and resulting in vasodilation, anti-inflammatory and antioxidant effects; (3) This process can be stimulated by the administration of rhsACE2 which inhibits the depletion of ACE2, or ACEIs/ARBs which block ACE/Ang II/AT1R pathway, resulting in increased ACE2 activation. *Ang II* angiotensin II, *ACE2* angiotensin-converting enzyme 2, *Ang 1–7* angiotensin 1–7, *ACEIs* angiotensin-converting-enzyme inhibitors, *ARBs* angiotensin II receptor blockers, *MasR* Mas receptor

The RAAS blockers inhibit the ACE activity and thus lead to the blockade of the ACE/AngII/AT1R pathway, and at the same time cause compensatory activation of the ACE2/Ang 1–7/MasR pathway, which has vasodilating, anti-inflammatory and antioxidant effects [71] (Fig. 2). In the course of SARS-CoV-2 infection, plasma Ang II levels were elevated and linearly dependent on viral load and severity of lung damage, as shown in the study by Liu et al. [72]. In contrast, a higher Ang1-7/Ang I ratio was observed in patients with acute respiratory distress syndrome (ARDS) who survived the infection than in non-survivors [73]. A study in mice showed that loss of ACE2 induced resistance to SARS-CoV-2 infection, but also increased vascular permeability, pulmonary edema, neutrophil accumulation, and lung dysfunction [30, 68]. It seems important that the use of recombinant ACE2 protects against acute lung injury in mouse models of ARDS [74, 75]. Also in clinical trials, rhsACE2 has been demonstrated to protect patients from lung damage [30, 76]. Therefore, administering rhsACE2 or increasing plasma sACE2 levels may become an interesting therapeutic strategy for SARS-CoV-2 inactivation [74].

Several large-number clinical trials [56, 77] have demonstrated the protective effect of ACEI/ARB in patients with COVID-19. The use of these drugs was associated with a milder course of the disease and a lower risk of death from any cause. A meta-analysis of 26 studies involving patients with hypertension confirmed such results [65].

The analysis of subsequent studies indicates the scarce relationship between the use of these drugs and the frequency of SARS-CoV-2 infection or the severity of COVID-19 infection. The use of ACEI/ARB is not associated with a higher probability of a positive test result for SARS-CoV-2 infection. Similar results were obtained in subgroups of older and younger patients. The course of infection was also gender-independent [78, 79]. The lack of effect of the discussed drugs on the course of COVID-19 infection was also noted in a large observational study by de Abajo et al. [80]. An interesting finding from this study is the lower risk of developing the COVID-19 infection requiring hospitalization in the diabetic subgroup taking ACEI/ARB. Diabetes has previously been reported as a risk factor for the severe course of COVID-19 [81], although the exact biological basis remains unknown. Experimental mice models of DM showed high ACE activity in the lungs [82].

To conclude, there is no evidence that drugs modulating the RAAS activity worsen the course of this infection [78–80]. Additionally, given that discontinuation of therapy with those drugs, regardless of comorbid infections, is detrimental to patients and worsens their prognosis, the said drugs – according to guidelines – should not be discontinued [78].

### ACE2 and thromboembolic events after COVID-19

**Fig. 3:**
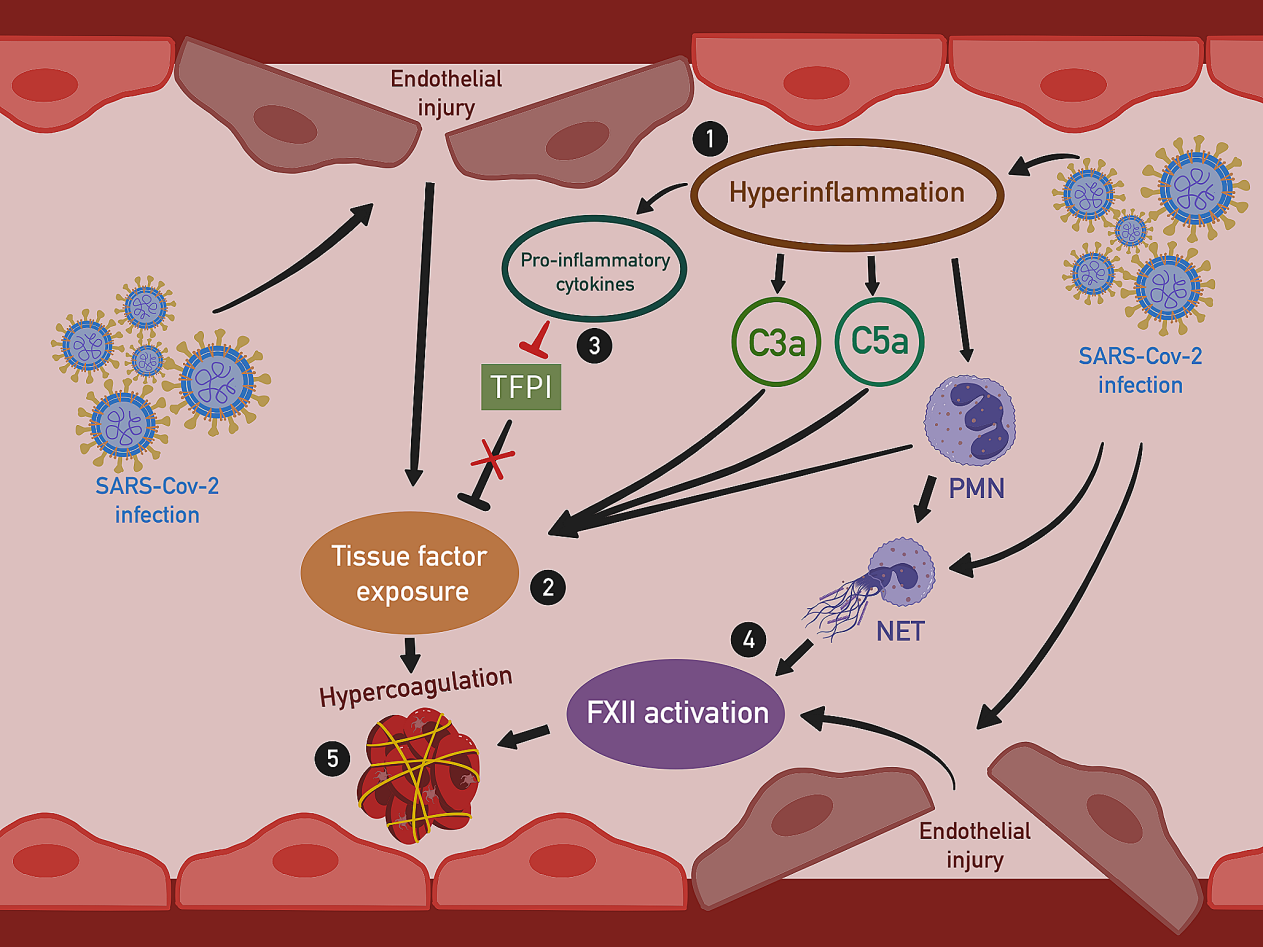
Mechanism of hypercoagulation in SARS-CoV-2 infection. (1) Hyperinflammation caused by SARS-Cov-2 infection leads to the production of C3a and C5a and the upregulation of PMNs; (2) Endothelial injury, PMNs, C3a and C5a increase TF exposure to blood coagulation factors; (3) Pro-inflammatory cytokines released in hyperinflammation inhibit TFPI; (4) SARS-CoV-2 infection leads to the release of neutrophil extracellular traps, which activate FXII. FXII is also activated by endothelial injury and PMNs; (5) Stimulation of both intrinsic coagulation pathway through FXII activation and extrinsic coagulation pathway through TF exposure results in hypercoagulation and prothrombotic state. *PMN* polymorphonuclear leukocytes, *TFPI* tissue factor pathway inhibitor, *FXII* coagulation factor XII, *C3a* complement component 3a, *C5a* complement component 5a

As a result of SARS-CoV-2 infection, endothelial dysfunction occurs through direct viral damage to endothelium as well as through the development of hyper-inflammation. Hypercoagulation development involves the activation of both intrinsic and extrinsic coagulation pathways. Activation of the internal coagulation pathway results from a loss of endothelial integrity and release of the neutrophil extracellular traps, activating coagulation factor XII (FXII). Furthermore, endothelial injury induced by the virus unveils tissue factor (TF) to coagulation factor VII (FVII) present in the blood, initiating thus the extrinsic coagulation pathway. Hyperinflammatory response to the infection leads to increased complement components 3a and 5a (C3a/C5a) activity that further stimulate TF exposure. In addition, the TF pathway inhibitor (TFPI) activity is reduced by pro-inflammatory cytokines. Moreover, polymorphonuclear leukocytes (PMNs) activate both internal and external coagulation pathways [83–85]. . (Fig. 3) Additionally, there is some evidence [86, 87] that SARS-CoV-2 may also activate platelets that are involved not only in thrombus formation but also in the release of sACE2. Finally, the effect of the RAAS on liver dysfunction in COVID-19, and thus on the deficiency of anticoagulant proteins and the increase in prothrombotic factors, seems to be an important contributor to the pathophysiology of thrombotic events [46].

**Fig. 4:**
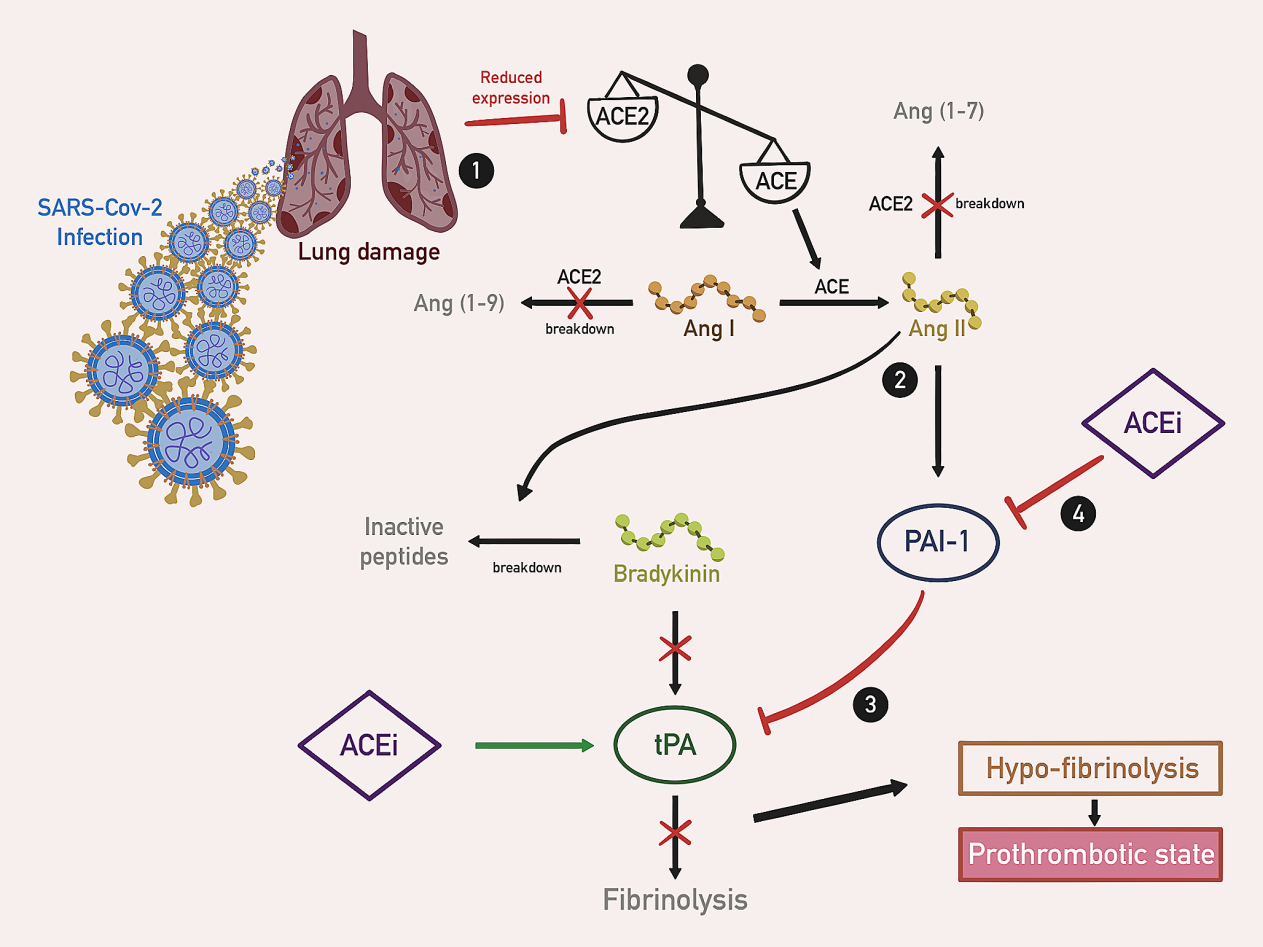
Mechanism of hypo-fibrinolysis in SARS-CoV-2 infection. (1) Lung damage in the course of the SARS-Cov-2 infection results in reduced ACE2 expression, leading to increased Ang II production by ACE; (2) Ang II induces expression of PAI-1 and the breakdown of bradykinin into inactive peptides. tPA production decreases due to bradykinin breakdown; (3) Excessive PAI-1 production further suppresses tPA activity, resulting in hypo-fibrinolysis and prothrombotic state; (4) Administration of ACEi has been suggested to decrease PAI-1 and increase tPA *Ang I* angiotensin I, *Ang II* angiotensin II, *Ang (1–7)* angiotensin (1–7), *Ang (1–9)* angiotensin (1–9), *ACE* angiotensin-converting enzyme, *ACE2* angiotensin converting enzyme 2, *tPA* tissue plasminogen activator, *ACEi* angiotensin-converting-enzyme inhibitors, *PAI-1* plasminogen activator inhibitor-1

It has been shown that the Ang II/AT1R axis, upregulated in the late stages of severe COVID-19, has prothrombotic activity [88]. Ang II stimulates plasminogen activator inhibitor-1 (PAI-1) secretion by endothelium and enhances the breakdown of bradykinin into inactive peptides [89, 90]. Bradykinin binds to the B2 bradykinin receptor (B2R) and stimulates the release of tissue-type plasminogen activator (tPA); thus bradykinin breakdown lowers tPA concentration [91]. As a result, elevated PAI-1 activity and reduced tPA levels impair fibrinolysis, promoting thrombus deposition in the alveoli and the small blood vessels [92, 93]. (Fig. 4)

On the other hand, the ACE2/Ang 1–7/MasR axis induced in the early phase of infection exerts an antithrombotic effect e.g. through the NO release from platelets [94, 95]. Interestingly, studies on rat models have shown that Ang 1–9 induces both platelet aggregation and impaired fibrinolysis [96, 97], thus acting synergistically with Ang II in inducing thrombotic/ischemic processes in severe forms of COVID-19.

Downregulation of the ACE2 by SARS-CoV-2 promotes hypo-fibrinolysis [98]. The prothrombotic state in COVID-19 results from both hypercoagulation and hypo-fibrinolysis. Under such conditions, immune factors and platelets form together an immuno-coagulant clot responsible for the thrombotic complications in the course of the disease [99]. While the administration of ACEIs has been linked to an increase in the tPA to PAI-1 ratio, the ARBs have not been proven to demonstrate such effects [98]. However, in mice model Spike-Fc-induced lung injury and edema were shown to be ameliorated by the blockade of AT1R with losartan [68].

Noteworthy, under hypoxic conditions in severe acute respiratory syndrome (SARS) both arms of the RAAS are activated. Consequently, there is a further induction of ACE2 expression on the surface of the cell membrane, generating the positive feedback that sustains COVID-19 infection [100].

### SARS-CoV-2 and the hyaluronic acid-induced inflammation - the role of the RAAS blockers

Interestingly, a recent case series has shown several cases of delayed inflammatory reaction to facial dermal hyaluronic acid filler which was following COVID-19 infection as well as in patients following the first dose of mRNA-1273 vaccine (Moderna, Cambridge MA), and after the second dose of BNT162b2 vaccine (Pfizer, New York, NY, U.S.). In all cases, the reaction occurred after a hyaluronic acid filler had been placed > 1 year before the vaccination. All patients responded rapidly to therapy with a low dose of ACEI, which decreased the cutaneous filler-related inflammatory reaction and edema. The authors postulate that, after vaccination, SARS-CoV-2 spike protein is produced extracutaneously and then, upon reaching the skin, binds to ACE2 receptors present on the cutaneous resident cells, including lymphocytes, fibroblasts, and adipocytes. Similarly to SARS-CoV-2 infection, this process leads to ACE2 internalization and down-regulation. As previously mentioned, ACE2 degrades Ang II to Ang 1–7, and its depletion causes a local inability to control Ang II concentration. The accumulation of Ang II up-regulates the expression of certain chemoattractants and activators of neutrophils, as well as the CD44 glycoprotein, which is capable of binding free extracellular hyaluronic acid, providing a link for inflammatory reaction targeted to the quiescent hyaluronic acid granuloma. The reduction of Ang II activity through the administration of ACEI may have resulted in a shift toward an anti-inflammatory response driven by Ang 1–7, thereby explaining the therapeutic effect of ACEI reported in this case series [101].

## Conclusions

The RAAS plays a critical role in the pathogenesis of hypercoagulation and hyperinflammation in the course of COVID-19. SARS-CoV-2 infection results in reduced expression of ACE2 and overactivation of ACE, shifting the balance between the ACE/Ang II/AT1R pathway and the ACE2/Ang 1–7/MasR pathway, towards the first one, which results in pro-inflammatory and pro-thrombotic effects that deteriorate patients’ prognosis. Some studies demonstrate promising results using particular RAAS components such as rhsACE2, sACE2-IgG conjugates as well as Zn^2+^ chelating agents, blocking the ADAM17 metalloproteinase. Also, a large body of evidence supports the recommendation to maintain the administration of ACEIs and ARBs, as they do not worsen the course of COVID-19 and may even exert a beneficial effect on the outcomes. Further studies are needed to better understand the role of the RAAS in the SARS-CoV-2 infection and to design novel therapeutic strategies for combating COVID-19.

## Data Availability

Not applicable.

## Abbreviations

ACE: Angiotensin-converting enzyme
ACE2: Angiotensin-converting enzyme 2
ACEIs: Angiotensin-converting enzyme inhibitors
ADAM17: A Disintegrin and Metalloproteinase 17
Ang I: Angiotensin I
Ang II: Angiotensin II
Ang 1-7: Angiotensin 1-7
Ang 1-9: Angiotensin 1-9
ARBs: Angiotensin AT1 receptor blockers
ARDS: Acute respiratory distress syndrome
ASCVD: Atherosclerotic cardiovascular disease
AT1R: Angiotensin type 1 receptor
AT2R: Angiotensin type 2 receptor
B2R: B2 bradykinin receptor
C3a: Complement component 3a
C5a: Complement component 5a
CKD: Chronic kidney disease
DM: Diabetes mellitus
EF: Ejection fraction
FXII: Coagulation factor XII
FVII: Coagulation factor VII
G-CSF: Granulocyte colony-stimulating factor
HF: Heart failure
HFmrEF: Heart failure with mid-range ejection fraction
HFrEF: Heart failure with reduced ejection fraction
IL: Interleukin
LC: Long COVID
mACE2: Membrane-bound ACE2
MasR: Mas receptor
PAI-1: Plasminogen activator inhibitor-1
PASC: Post-acute sequelae of SARS-CoV-2 infection
PMNs: Polymorphonuclear leukocytes
POTS: Postural orthostatic tachycardia syndrome
RAAS: Renin-angiotensin-aldosterone system
RBD: Receptor-binding domain
rhsACE2: Recombinant human soluble ACE2
S: Spike protein
sACE2: Soluble ACE2
SARS: Severe acute respiratory syndrome
Sirt1: Sirtuin 1
TF: Tissue factor
TFPI: Tissue factor pathway inhibitor
TIMP3: Tissue inhibitor of metalloproteinase 3
TMPRSS2: Cellular transmembrane protease serine 2
TNF-alpha: Tumour necrosis factor-alpha
tPA: Tissue-type plasminogen activator
WHO: World Health Organization
